# Integrin-linked kinase mRNA expression in circulating mononuclear cells as a biomarker of kidney and vascular damage in experimental chronic kidney disease

**DOI:** 10.1186/s12964-024-01646-2

**Published:** 2024-05-11

**Authors:** Sofía Campillo, Elena Gutiérrez-Calabrés, Susana García-Miranda, Mercedes Griera, Loreto Fernández Rodríguez, Sergio de Frutos, Diego Rodríguez-Puyol, Laura Calleros

**Affiliations:** 1 Department of Systems Biology, Physiology Unit, Universidad de Alcalá, Alcalá de Henares, Madrid, Spain; 2 Department of Medicine and Medical Specialties, Universidad de Alcalá, Alcalá de Henares, Madrid, Spain; 3 Fundación Renal Iñigo Álvarez de Toledo (FRIAT), Instituto Ramón y Cajal de Investigación Sanitaria (IRYCIS), and RICORS2040 Kidney Disease, Instituto de Salud Carlos III, Madrid, Spain; 4 Biomedical Research Foundation and Nephrology Unit, Hospital Universitario Príncipe de Asturias, Alcalá de Henares, Madrid, Spain

**Keywords:** ILK, Biomarker, Renal damage, Vascular damage, Chronic kidney disease (CKD)

## Abstract

**Background:**

Traditional biomarkers of chronic kidney disease (CKD) detect the disease in its late stages and hardly predict associated vascular damage. Integrin-linked kinase (ILK) is a scaffolding protein and a serine/threonine protein kinase that plays multiple roles in several pathophysiological processes during renal damage. However, the involvement of ILK as a biomarker of CKD and its associated vascular problems remains to be fully elucidated.

**Methods:**

CKD was induced by an adenine-rich diet for 6 weeks in mice. We used an inducible ILK knockdown mice (cKD-ILK) model to decrease ILK expression. ILK content in mice's peripheral blood mononuclear cells (PBMCs) was determined and correlated with renal function parameters and with the expression of ILK and fibrosis and inflammation markers in renal and aortic tissues. Also, the expression of five miRNAs that target ILK was analyzed in whole blood of mice.

**Results:**

The adenine diet increased ILK expression in PBMCs, renal cortex, and aortas, and creatinine and urea nitrogen concentrations in the plasma of WT mice, while these increases were not observed in cKD-ILK mice. Furthermore, ILK content in PBMCs directly correlated with renal function parameters and with the expression of renal and vascular ILK and fibrosis and inflammation markers. Finally, the expression of the five miRNAs increased in the whole blood of adenine-fed mice, although only four correlated with plasma urea nitrogen, and of those, three were downregulated in cKD-ILK mice.

**Conclusions:**

ILK, in circulating mononuclear cells, could be a potential biomarker of CKD and CKD-associated renal and vascular damage.

**Graphical Abstract:**

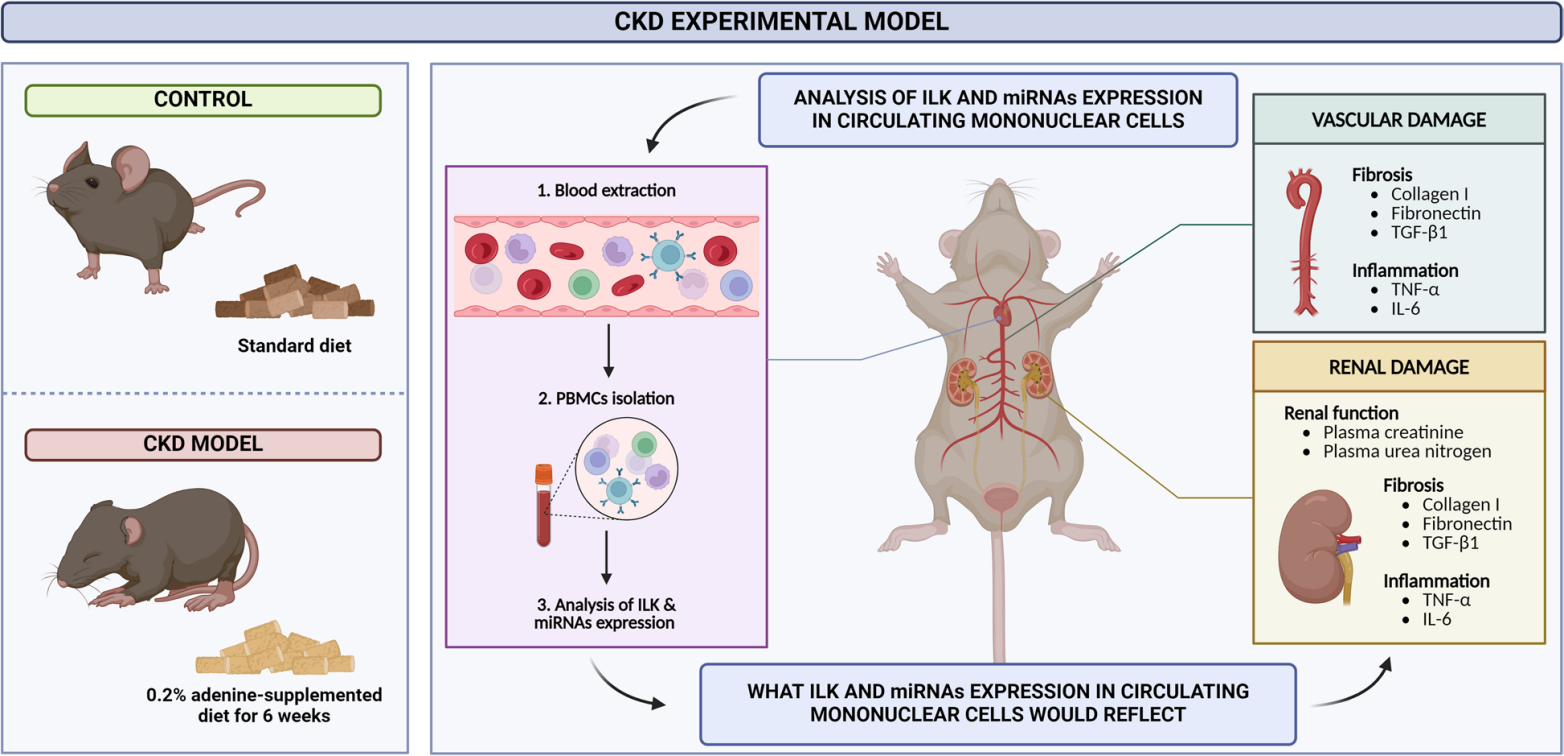

ILK content in circulating mononuclear cells reflects renal and vascular damage in a CKD experimental model.

## Background

Chronic kidney disease (CKD) is defined by a glomerular filtration rate (GFR) under 60 ml/min/1.73m^2^ or markers of kidney damage for at least 3 months [1, 2]. Globally, more than 850 million individuals had kidney diseases in 2017 [3], and 1.2 million people died from CKD the same year [4]. CKD is reaching epidemic levels due to the increasing prevalence of diabetes mellitus, hypertension, and obesity, as well as aging of the population. The Global Burden of Diseases, Injuries, and Risk Factors Study projected that CKD will rise in ranking from the 16th global cause of death in 2016 to the 5th position in 2040 [5]. Additionally, CKD has a significant socioeconomic impact on society. The extrapolated annual cost of all CKD is at least as high as that for cancer or diabetes, even more so if indirect costs related to cardiovascular complications are included [6].

Nowadays, the link between CKD and cardiovascular disease (CVD) is indisputable and CVD mortality is significantly higher in patients with CKD than in the general population. Accumulation of uremic toxins leads to systemic inflammation, which is a key predictor of cardiovascular events and death in CKD, and vascular calcification and arterial stiffness are major contributors to this elevated mortality [7]. Due to the process of cardiovascular damage starts very early during progression, earlier identification and treatment of CVD in patients with CKD may reduce the severity of the disease and improve the outcomes of those who reach end-stage renal disease treatment. The trouble with the diagnosis of CKD is that most patients are asymptomatic in the early stages of the disease and its course is difficult to predict.

In clinical practice, CKD is diagnosed based on measurements of GFR, creatinine, cystatin C, blood urea nitrogen, and albuminuria, which do not detect CKD in the early stages nor predict the course of the disease [8]. Furthermore, the utility of these traditional biomarkers for cardiovascular prediction is controversial [9]. On the other hand, renal biopsy is an essential procedure in the diagnosis of renal diseases, although it carries the risk of major complications [10], such as bleeding and renal loss. Many reviews have focused on the research of new non-invasive biomarkers of CKD and vascular pathologies in CKD [11], including miRNAs, endogenous non-coding RNA molecules that regulate gene expression by repressing the translation and/or inducing the degradation of their mRNA targets [12, 13]. However, verification, validation, and commercial development of novel biomarkers is an immensely expensive and high-risk process.

Integrin-linked kinase (ILK) is a scaffolding protein and a serine/threonine protein kinase that plays multiple roles in regulating integrin-mediated processes such as cell adhesion, survival, proliferation, migration, and extracellular matrix (ECM) deposition [14]. Both upregulation and downregulation of ILK expression and/or activity have been implicated in the pathogenesis of a wide variety of diseases such as diabetes [15], myocardial infarction [16], obesity [17] or pulmonary hypertension [18], suggesting ILK as a molecular target and a prognostic biomarker of these diseases. Several publications, including ours, have described ILK as a mediator in different pathological processes that occur during renal damage, including inflammatory responses, epithelial-mesenchymal transition, and fibrosis [19–21]. Also, we have demonstrated that ILK depletion in vivo prevents CKD progression induced by an adenine-rich diet [22]. Regarding vascular physiologic and pathologic processes, in vivo and in vitro models showed that ILK plays a critical role in regulating vascular tone [23, 24], and leucocyte adhesion to the endothelium [25]. Recently, our group has demonstrated that ILK is required for the formation of podosomes, structures that mediate cell adhesion and migration of monocytes across ECM-based barriers [26], which cause dysfunction and vascular damage in the context of uremia.

The finding of new non-invasive biomarkers would improve our ability to diagnose CKD and its vascular-associated damage earlier and maybe predict its progression. Therefore, the aim of this study is to investigate the potential of ILK content in circulating mononuclear cells as a biomarker of CKD-associated renal and vascular damage in the context of experimental CKD. Additionally, the possibility that some miRNAs involved in the regulation of ILK could constitute alternative biomarkers to ILK itself was also tested.

## Methods

### Animal study design

All procedures involving animals were previously approved by the Institutional Animal Care and Use Committee of the University of Alcalá and conformed to Directive 2010/63/EU of the European Parliament. Animals were housed in a pathogen-free and temperature-controlled room (22 °C ± 2 °C). Food and water were available ad libitum.

CKD was induced in mice by feeding a diet containing 0.2% adenine (Sigma, St Louis, MO, USA) as previously described [22]. To decrease ILK expression, we used an inducible ILK knockdown mice (cKD-ILK) model, explained in prior publications [22, 26]. Briefly, conditional inactivation of the ILK gene was accomplished by crossing C57Bl/6 mice homozygous for the floxed ILK allele (LOX mice) with homozygous mice carrying a tamoxifen-inducible CreER(T) recombinase gene, which express Cre under the control of the cytomegalovirus promoter (CRE mice). Tamoxifen was dissolved in a corn oil/ethanol (9:1) mixture. Male CRE-LOX mice (8-week-old), heterozygous for both transgenes, were injected intraperitoneally with 1.5 mg of tamoxifen once per day for 5 consecutive days to induce ILK depletion. Control animals were injected with the vehicle, a corn oil/ethanol (9:1) mixture. Three weeks after the injections, tail DNA was genotyped by PCR with primers allow to distinguish excised ILK gene (230 bp) or non-excised ILK (2100 bp): CCAGGTGGCAGAGGTAAGTA and CAAGGAATAAGGTGAGCTTCAGAA [22, 26]. PCR DNA products were then analyzed by 1.5% agarose gel electrophoresis. Tamoxifen-treated CRE-LOX mice displaying successful depletion of ILK were termed cKD-ILK mice, and their control vehicle-treated CRE-LOX mice were termed wild-type (WT). These animals were also fed with the adenine diet. After 6 weeks, mice were anesthetized, and peripheral blood was extracted intracardially and collected in tubes with 0.1% EDTA as anticoagulant. Plasma was separated by centrifugation at 3000 rpm for 15 min and stored at − 80 °C until assayed. Peripheral blood mononuclear cells (PBMCs) were isolated by Ficoll-Paque density gradient centrifugation (Lymphocytes Isolation Solution, Rafer, UK). Renal cortex (RC) and aortas were collected and, together with the PBMCs, they were stored in RNAlater solution (Life Technologies, Carlsbad, CA, USA) at − 80ºC for the RT-qPCR assays.

### Renal function measurements

Plasma creatinine (700,460; Cayman Chemical; Ann Arbor, MI, USA) and urea nitrogen (EIABUN; Invitrogen, Thermo Fisher Scientific; Waltham, MA, USA) were measured using colorimetric assay kits, according to the manufacturer’s instructions. The spectrophotometric measurements were performed in a Victor X4 Multilabel Plate Reader (PerkinElmer, Waltham, MA, USA) at a wavelength of 490 nm (plasma creatinine) and 450 nm (plasma urea nitrogen).

### Reverse transcription– quantitative polymerase chain reaction (RT-qPCR)

Total RNA of each sample was extracted with TRIzol, transcribed into cDNA with a High-Capacity cDNA Reverse Transcription Kit (Life Technologies, Carlsbad, CA, USA) and RT-qPCR analysis was performed in a 7500 qPCR thermocycler. The normalized gene expression method (2^–ΔΔCT^) for relative quantification of gene expression was used [22].

Non-excised ILK mRNA levels were measured in mice PBMCs, renal cortex (RC) and aortas by RT-qPCR with SYBR Green Master Mix to verify that ILK depletion had also occurred in these cells and tissues [22, 26]. Primers GGGCTCTTGTGAGCTTCTGT and GAGTGGTCCCCTTCCAGAAT, designed to recognize the cDNA sequence between exons within floxed areas 6 and 7 [22, 26] were used. For the rest of the genes, RT-qPCR with SYBR Green Master Mix were performed using primers designed in the PUBMED Gene Database: collagen I (COL I), 5′-TCCTGGCAACAAAGGAGACA-3′ (forward) and 5′-GGGCTCCTGGTTTTCCTTCT-3′ (reverse); fibronectin (FN), 5′-TGAGCGCCCTAAAGATTCCA-3′ (forward) and 5′-TAGCCACCAGTCTCATGTGC-3′ (reverse); TGF-β1, 5′-TTGCTTCAGCTCCACAGAGA-3′ (forward) and 5′-TGGTTGTAGAGGGCAAGGAC-3′ (reverse); TNF-α, 5′- TGGCCCAGACCCTCACACTCA-3′ (forward) and 5′-GGCTCAGCCACTCCAGCTGC-3′ (reverse); IL-6, 5′-CCGGAGAGGAGACTTCACAGAGGA-3′ (forward) and 5′- AGCCTCCGACTTGTGAAGTGGTATA-3′ (reverse); β-actin, 5′-GACGGCCAGGTCATCACTAT-3′ (forward) and 5′-CTTCTGCATCCTGTCAGCAA-3′ (reverse).

### miRNA search and extraction, and RT-qPCR

To investigate novel potent biomarkers in the whole blood of mice fed a diet rich in adenine, we performed bioinformatic analysis in different miRNAs-specific databases. We searched which miRNAs target ILK in TargetScan (https://www.targetscan.org/vert_80/) and miRbase databases (https://www.mirbase.org/) and which of them were common between mice and humans, which is important when establishing interspecies parallels. Then, we investigated which of these miRNAs were more expressed in kidney in https://ccb-web.cs.uni-saarland.de/tissueatlas [27] and, finally, we selected 5 miRNAs: miR-542-3p, miR-758-3p, miR-361-3p, miR-30c-1-3p and miR-30c-2-3p. The binding sites of miRNAs in the ILK 3’-UTR region were predicted via TargetScan database.

To analyze the expression of miRNAs in whole blood of mice, peripheral blood was extracted intracardially and collected in tubes of PAXgene Blood RNA (BD Biosciences; San Jose, CA, USA). Extraction of miRNAs was performed using the QIAcube Connect (Qiagen; Venlo, Netherlands), following manufacturer’s instructions. Concentration of miRNAs was determined in a spectrophotometer (NanoDrop) and miRNAs were transcribed into cDNA with the miRCURY LNA RT kit (Qiagen; Venlo, Netherlands). RT-qPCRs were carried out using primers against miR-542-3p, miR-758-3p, miR-361-3p, miR-30c-1-3p and miR-30c-2-3p, and with the miRCURY LNA SYBR Green PCR kit (Qiagen; Venlo, Netherlands). miR-103a-3p was used as endogenous control. The analysis was performed in a 7500 qPCR thermocycler and the normalized gene expression method (2^–ΔΔCT^) for relative quantification of gene expression was used [22].

### Statistical analysis

All the data were analyzed with the GraphPad Prism software (La Jolla, CA, USA). The results are expressed as the mean ± SEM. As the number of animals or samples in the different experiments was never over 10, non-parametric statistics were used for comparisons, applying the Kruskal–Wallis test with Mann–Whitney post-test (non-paired data) or the Friedman test with Wilcoxon post-test (paired data). In both cases, Bonferroni correction was used. Correlation analysis was performed using linear regression for each genotype (combining WT Control, cKD-ILK Control, WT Adenine, and cKD-ILK Adenine treatments) and plotted on the same graph. A *p* value < 0.05 was considered statistically significant.

## Results

### ILK content in PBMCs correlates directly with renal function and ILK expression in renal cortex (RC) and aorta of mice with experimental CKD

**Fig. 1:**
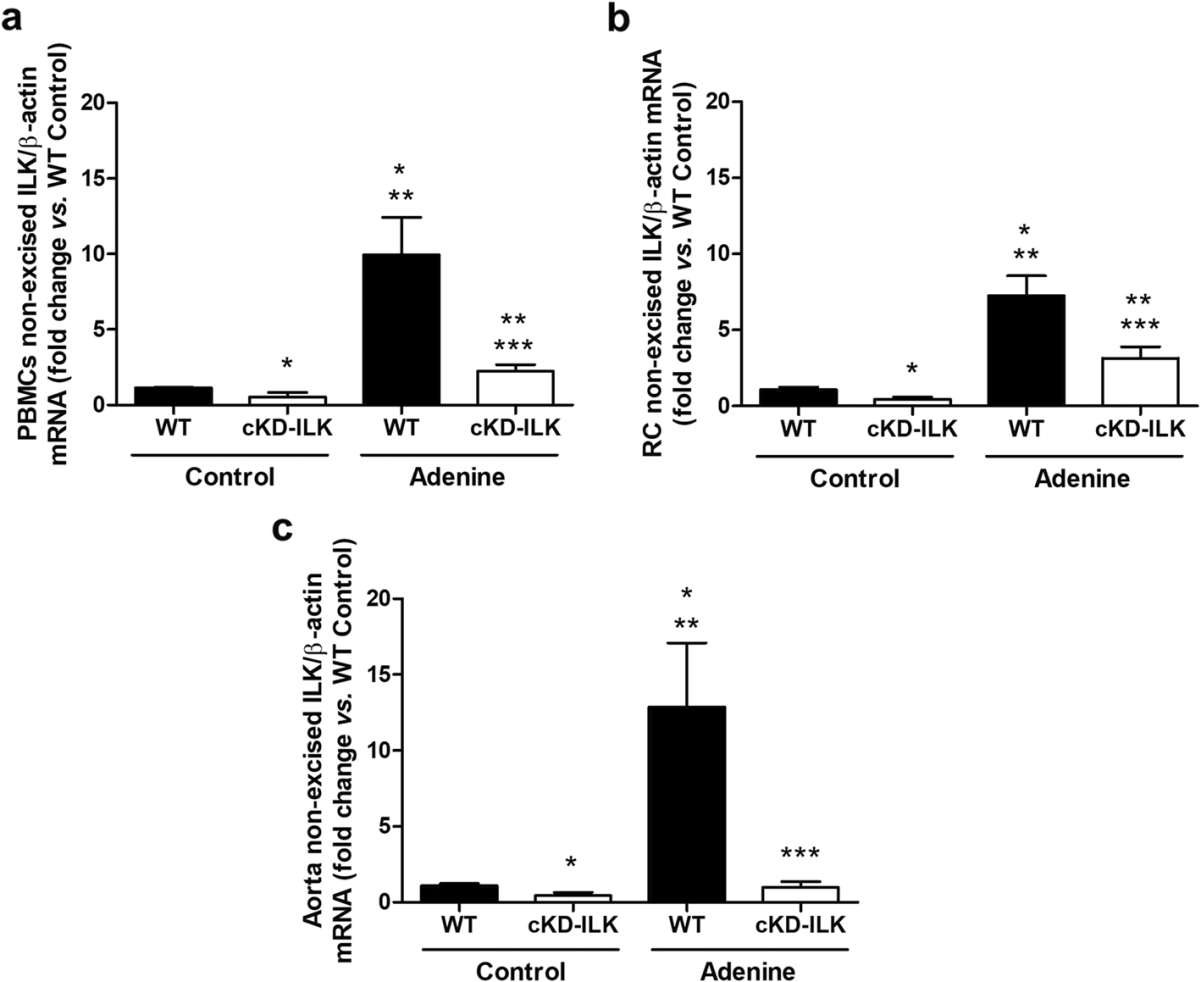
ILK content increases in PBMCs, RC, and aorta of adenine-fed mice. Wild-type (WT) and ILK conditional-knockdown (cKD-ILK) mice were fed a standard (Control) or an adenine-rich (Adenine) diet for 6 weeks. Non-excised ILK mRNA expression in peripheral blood mononuclear cells (PBMCs) (**a**), renal cortex (RC) (**b**), and aorta (**c**), normalized against β-actin as the endogenous control, was measured. Results are shown as mean ± SEM. *p < 0.05 *vs.* WT Control; **p < 0.05 *vs*. cKD-ILK Control; ***p < 0.05 *vs.* WT Adenine. This experimental design was replicated in two cohorts with sample sizes of 15 and 13 animals, respectively. *n* = 3–9 animals/group

To investigate the possible relationship between ILK content in PBMCs and renal function, we measured creatinine and urea nitrogen in the plasma of WT and cKD-ILK mice fed standard or adenine-rich diet for 6 weeks. As we previously published [22], plasma creatinine and plasma urea nitrogen were significantly higher in mice fed adenine-rich diet, compared to mice fed standard diet, while this increase was significantly lower in cKD-ILK mice (Table 1). Then, we analyzed the expression of ILK in PBMCs, RC, and aortas of the same animals. These results showed a statistically significant increase in non-excised ILK mRNA expression in PBMCs, RC (as we previously published in [22]) and aortic tissues of mice fed the adenine-rich diet, compared to mice fed the standard diet, while ILK transgenic depletion prevented the ILK expression increase in cKD-ILK mice (Fig. 1).

**Table 1:** Renal function parameters of wild-type (WT) and ILK conditional-knockdown (cKD-ILK) mice

Groups	WT Control	cKD-ILK Control	WT Adenine	cKD-ILK Adenine
Plasma Parameters				
Creatinine (mg/dl)	0.26 ± 0.01	0.31 ± 0.01	0.86 ± 0.04^*,**^	0.35 ± 0.01^*,***^
Urea nitrogen (mg/dl)	21.4 ± 2.1	21.6 ± 3.0	98.8 ± 14.9^*,**^	40.1 ± 9.8^***^

Renal function was assessed by measuring plasma creatinine and urea nitrogen concentrations of WT and cKD-ILK mice fed a standard (Control) or an adenine-rich (Adenine) diet for 6 weeks. Results are shown as mean ± SEM

^*^p < 0.05 *vs.* WT Control

^**^p < 0.05 *vs.* cKD-ILK Control

^***^p < 0.05 *vs*. WT Adenine. This experiment was made in only one cohort of 18 animals. *n* = 3–6 animals/group

**Fig. 2:**
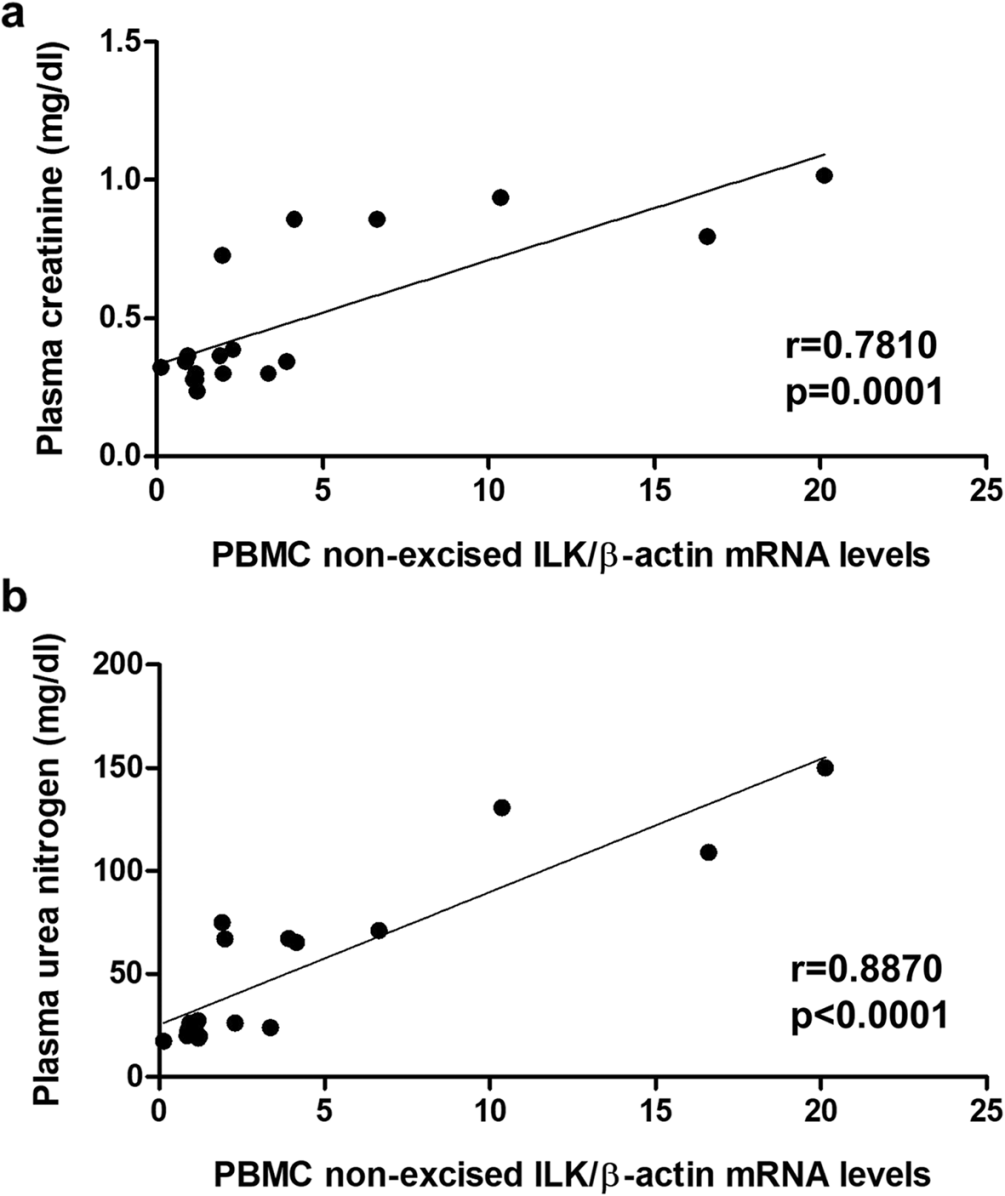
ILK content in PBMCs of mice correlates with renal function parameters. Wild-type and ILK conditional-knockdown mice were fed a standard or an adenine-rich diet for 6 weeks. ILK mRNA expression in peripheral blood mononuclear cells (PBMCs) was confronted with the values of plasma creatinine (**a**) and plasma urea nitrogen (**b**) (mg/dl). This experiment was made in only one cohort of 18 animals. *n* = 3–6 animals/group. The analysis is detailed in the Methods section

**Fig. 3:**
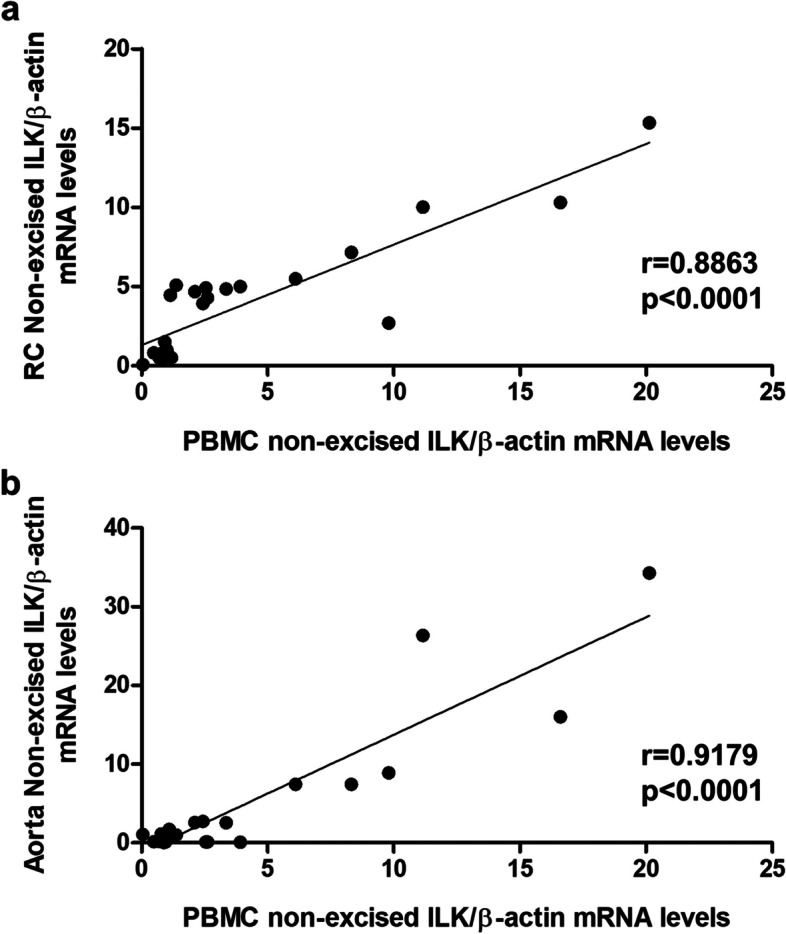
ILK content in PBMCs correlates with ILK content in RC and aorta of mice. Wild-type and ILK conditional-knockdown mice were fed a standard or an adenine-rich diet for 6 weeks. ILK mRNA expression in peripheral blood mononuclear cells (PBMCs) was confronted with the values of ILK mRNA expression in renal cortex (RC) (**a**) and aorta (**b**) of mice. This experimental design was replicated in two cohorts with sample sizes of 15 and 13 animals, respectively. *n* = 4–9 animals/group. The analysis is detailed in the Methods section

Given that with these results we obtained a wide range of ILK concentrations in PBMCs and in renal and aortic tissues, we proceeded to correlate the ILK content in PBMCs with plasma creatinine (Fig. 2a) and urea nitrogen (Fig. 2b). Interestingly, a statistically significant direct correlation was shown with both renal function parameters. In addition, our results showed a statistically significant direct correlation between ILK mRNA expression in PBMCs and in RC (Fig. 3a) and aorta (Fig. 3b), revealing a highly significant relationship between circulating and local ILK content.

### ILK content in PBMCs reflects renal and vascular damage in a CKD experimental model

**Fig. 4:**
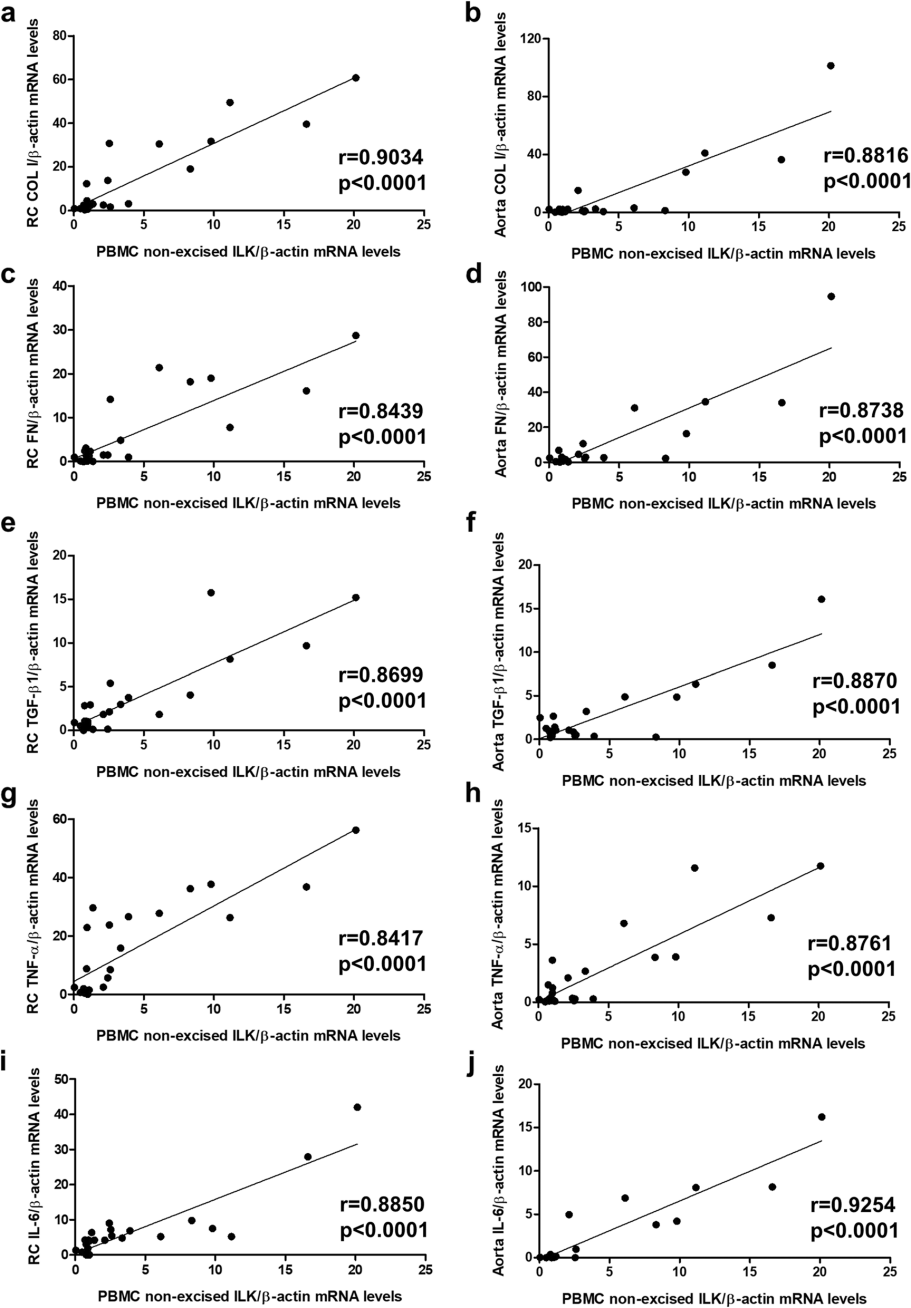
ILK content in PBMCs correlates with fibrosis and inflammation marker expression in RC and aorta. Wild-type and ILK conditional-knockdown mice were fed a standard or an adenine-rich diet for 6 weeks. ILK mRNA expression in peripheral blood mononuclear cells (PBMCs) was confronted with the values of collagen I (COL I) (**a**, **b**), fibronectin (FN) (**c**, **d**), TGF-β1 (**e**, **f**), TNF-α (**g**, **h**), and IL-6 (**i**, **j**) mRNA expression in renal cortex (RC) (**a**, **c**, **e**, **g**, **i**) and aorta (**b**, **d**, **f**, **h**, **j**) of mice. This experimental design was replicated in two cohorts with sample sizes of 15 and 13 animals, respectively. *n* = 4–8 animals/group. The analysis is detailed in the Methods section

To test whether ILK content in PBMCs may also reflect renal and vascular fibrosis and inflammation, both considered pathogenic events that characterize organ damage and, in some cases, disease progression, we analyzed the correlations between ILK content in PBMCs and fibrosis markers and inflammatory cytokines in mice renal and aortic tissues. Our results showed a statistically significant direct correlation between ILK in PBMCs and the expression of collagen I (Fig. 4a-b), fibronectin (Fig. 4c-d), the profibrotic cytokine TGF-β1 (Fig. 4e-f), TNF-α (Fig. 4g-h), and IL-6 (Fig. 4i-j) both in RC and aortas.

### The expression of miRNAs that target ILK correlates directly with renal function in a CKD experimental model

**Fig. 5:**
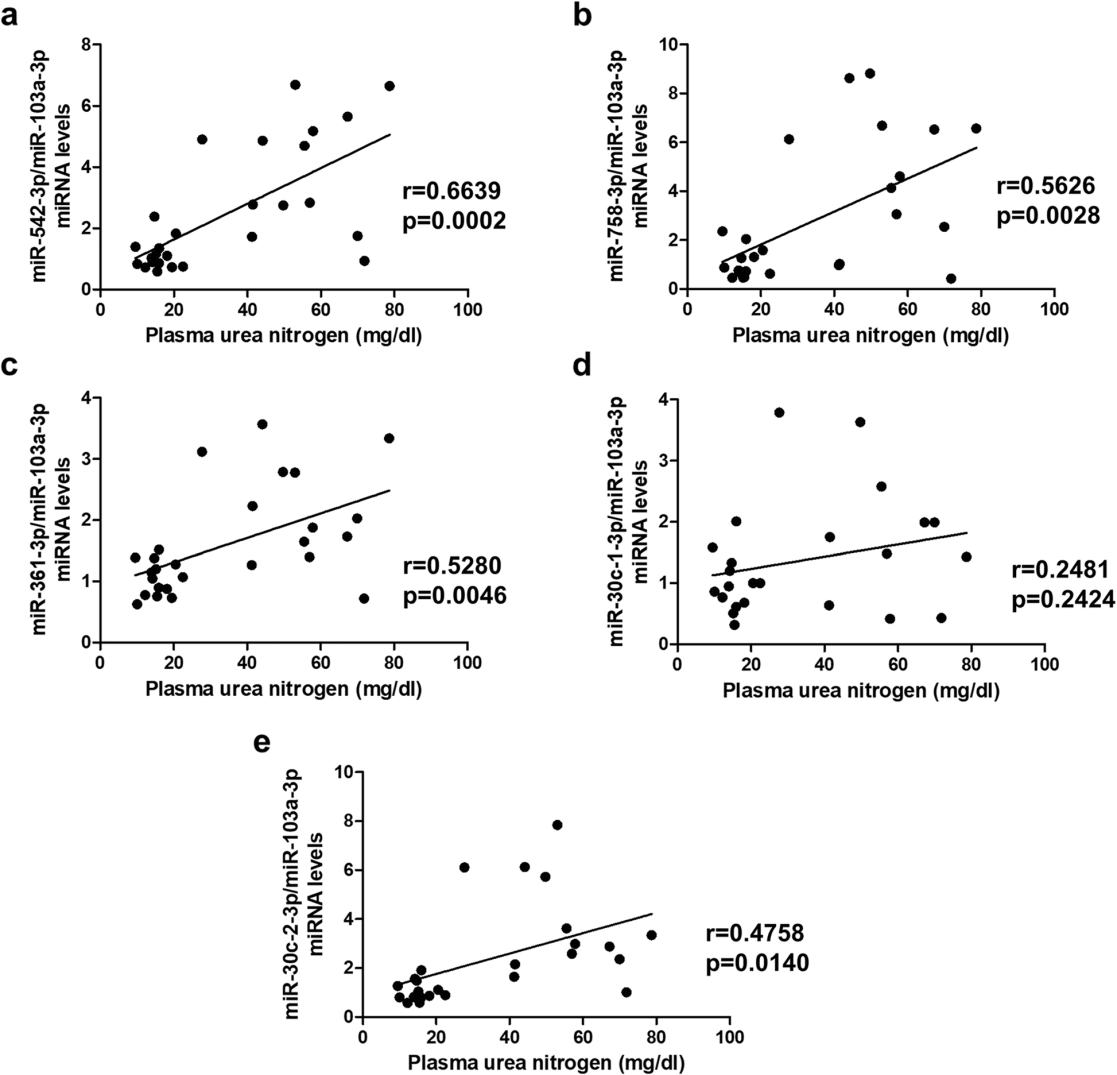
miRNAs content in whole blood of mice correlation with plasma urea nitrogen. Wild-type and ILK conditional-knockdown mice were fed a standard or an adenine-rich diet for 6 weeks. Plasma urea nitrogen (mg/dl) measurements were confronted with the values of miR-542-3p (**a**), miR-758-3p (**b**), miR-361-3p (**c**), miR-30c-1-3p (**d**) and miR-30c-2-3p (**e**) in whole blood of mice. This experimental design was replicated in three cohorts with sample sizes of 7, 10, and 10 animals, respectively. *n* = 3–11 animals/group. The analysis is detailed in the Methods section

After searching different miRNA-specific databases (see Methods section), five miRNAs that target ILK were selected as possible potent biomarkers of CKD in whole blood of mice: miR-542-3p, miR-758-3p, miR-361-3p, miR-30c-1-3p, and miR-30c-2-3p. Interestingly, a significant increase in the levels of all five miRNAs in the whole blood of adenine-fed mice was observed compared to controls (Table 2). However, a statistically significant direct correlation only was observed between plasma urea nitrogen and the expression of miR-542-3p, miR-758-3p, miR-361-3p, and miR-30c-2-3p (Fig. 5).

**Table 2:** MiRNAs content in whole blood of wild-type (WT) and ILK conditional-knockdown (cKD-ILK) mice

Groups	WT Control	cKD-ILK Control	WT Adenine	cKD-ILK Adenine
miRNAs				
miR-542-3p	1.0 ± 0.4	1.4 ± 0.9	4.2 ± 1.9^*^	3.8 ± 2.0^*^
miR-758-3p	1.1 ± 0.7	0.7 ± 0.5	6.5 ± 2.5^*,**^	3.4 ± 2.5^*,**,***^
miR-361-3p	1.0 ± 0.3	1.1 ± 0.3	3.0 ± 0.6^*,**^	1.7 ± 0.6^*,***^
miR-30c-1-3p	1.1 ± 0.4	0.7 ± 0.5	2.7 ± 1.2^*,**^	1.3 ± 0.8^***^
miR-30c-2-3p	1.1 ± 0.4	1.0 ± 0.5	4.7 ± 1.8^*,**^	2.4 ± 0.9^*,**^

MiRNA expression in whole blood of WT and cKD-ILK mice fed a standard (Control) or an adenine rich (Adenine) diet for 6 weeks was measured by RT-qPCR and normalized against miR-103a-3p as endogenous control. Results are shown as mean ± SEM

^*^p < 0.05 *vs*. WT Control

^**^p < 0.05 *vs.* cKD-ILK Control

^***^p < 0.05 *vs.* WT Adenine. This experimental design was replicated in three cohorts with sample sizes of 7, 10 and 10 animals, respectively. *n* = 3–11 animals/group

## Discussion

The main finding of the present work was that ILK content in circulating mononuclear cells strongly correlates with ILK content, and fibrosis and inflammation markers, in kidneys and aortas. Correlation coefficients obtained for the different analysis performed ranged between 0.84 and 0.93, with statistical significances always under 0.0001, suggesting that ILK content in circulating mononuclear cells reflects rather well the pathological changes that take place at renal and vascular levels in CKD.

The discovery of new potential biomarkers will provide non-invasive and safe approaches to obtaining relevant tools for the early diagnosis and prognosis of CKD patients [28]. Several investigations have identified ILK as a biomarker, especially in cancer since its involvement in migration and invasion processes [29, 30]. However, to the best of our knowledge, no study has ever reported whether ILK could be useful as a CKD biomarker despite its elevated expression levels observed in a wide variety of renal diseases [31–33]. To test this possibility, we used animals in which we could achieve a wide range of ILK concentrations. Advanced CKD was caused by the administration of high amounts of adenine in the diet for 6 weeks, which induces a profile of tubulointerstitial damage similar to that seen in human CKD. These animals exhibited increased plasma creatinine (approximately 3.3-fold) and urea nitrogen (approximately 4.5-fold) concentrations, as well as increased kidney ILK contents [22]. ILK depletion in mice was achieved with an inducible knockdown ILK global model, previously described by our group, which was used to demonstrate the role of ILK in the genesis of chronic renal damage [22]. Present experiments confirm published results, with changes in ILK renal content comparable to those previously observed, increased content in adenine-fed mice and decreased content in knockdown animals, as well as adenine-induced renal dysfunction that was prevented by ILK depletion. Additionally, an increased ILK content was also observed in vascular walls.

The good correlations between ILK content in circulating mononuclear cells and the renal function parameters tested in this study support the hypothesis that ILK can be considered a good biomarker of renal dysfunction. However, there are already other biomarkers, such as creatinine, blood urea nitrogen, cystatin, or albuminuria that detect CKD even in the early stages of diseases and are routinely performed in clinical practice [8]. For this reason, we investigated whether ILK could serve as a biomarker of renal and vascular tissue damage. Renal fibrosis is one of the most important pathological processes of CKD and its deregulation is decisive to lead to renal failure [34, 35], while renal inflammation is an essential pathological change that is closely associated with the development of renal and vascular damage [36]. Our excellent correlations between the ILK content in circulating mononuclear cells and the expression of fibrosis and inflammation markers, in addition to its own expression, in kidneys and aortas demonstrate the relevance of mononuclear cell ILK content as a reflection of what occurs in these tissues. The reason why ILK increases in circulating mononuclear cells when renal and vascular damage occurs in CKD is not yet clear. Several studies corroborate the hypothesis whereby the accumulation of uremic toxins in the organism of CKD patients is involved in the development of CKD-related cardiovascular damage, systemic inflammation, and immune deficiency [37–40]. In this regard, our group demonstrated that ILK activity is involved in monocyte adhesion and migration induced by uremic toxins [26], which might cause endothelial dysfunction. Therefore, uremia could probably be the reason why ILK expression is increased in circulating mononuclear cells during CKD. However, our in vitro experiments did not show an increase in ILK content of monocytes treated with uremic toxins [26], perhaps due to the low percentage of monocytes in blood compared to other types of circulating mononuclear cells. Altogether, these results demonstrate that ILK detection in circulating mononuclear cells could indeed be a useful method to detect renal and vascular fibrosis and inflammation, without the need for invasive or expensive methods method such as renal biopsy or vascular imaging. Adequately tested in human cohorts, the measurement of ILK content in mononuclear circulating cells could become a valuable prognostic biomarker, as it informs about the current degree of tissue damage.

Most of the molecules tested as possible biomarkers in CKD have been evaluated in blood and urine samples [8, 41]. Even though both blood and urine samples are promising, some reviews propose that urine biomarkers are better at predicting a rapid decline of renal function and CKD diagnosis compared with blood biomarkers [8]. For example, urinary neutrophil gelatinase–associated lipocalin (NGAL) levels in humans correlated with renal dysfunction, interstitial fibrosis, and tubular atrophy [42]. Kidney injury molecule-1 (KIM-1) urinary excretion has been shown to be a highly sensitive and specific marker of acute kidney injury [43]. The decline in serum and urinary Klotho concentration has been identified as an early CKD biomarker, and it is able to predict cardiovascular risk [44]. Furthermore, studies based on the measurement of estimated glomerular filtration rate and albuminuria have demonstrated that these parameters independently predict cardiovascular risk [9]. In contrast, the information concerning experimental approaches based on the use of circulating cellular biomarkers in CKD is scarce, and our study supports the potential applications of this strategy.

On the other hand, many works have described the role of different miRNAs in renal diseases, both as therapeutic targets and as biomarkers [45, 46]. The fact that miRNAs can be detected in biofluids, such as blood and urine, as part of protein complexes or in extracellular vesicles, can be useful to investigate their potential as biomarkers in many diseases including CKD in a non-invasive way [47]. In our case, we decided to use the whole blood of the mice to analyze the expression of different miRNAs that target ILK, allowing us to access both circulating mononuclear cells (just as ILK was analyzed) and extracellular organelles. To select our study miRNAs, we reviewed the publications that show which miRNAs have ILK as a target. ILK has been determined as the target of miR-542-3p and miR-625-3p in different pathologies, especially cancer [48, 49]. However, since miR-625-3p is not conserved between mice and humans according to the TargetScan database, we discarded it for our study. The rest of the miRNAs were selected as we explain in the Methods section. Additionally, the potential biological relevance of some of these miRNAs was also supported by experimental evidence. MiR-542-3p was found to be highly expressed in an in vitro model based on the high glucose treatment on the HK-2 cells [50]. In renal tissues of in vivo models, both miR-542-3p and miR-361-3p expressions were increased in a rat model of unilateral ureteral obstruction [50, 51], while miR-30c expression was significantly decreased in a mice model of diabetic nephropathy [52]. However, miR-30c-2-3p expression was increased under hypertonicity in KC3AC1 cells from mouse kidney cortical collecting ducts and in the kidneys of a hypertonicity mice model [53]. Conversely, miR-542-3p expression was decreased in the aortas of a rat model of 5/6 nephrectomy [54]. Furthermore, miR-758-3p has been studied as potential diagnostic biomarker of lupus nephritis in plasma samples from patients with systemic lupus erythematosus [55]. All these studies show that these miRNAs might play an important role in CKD and that they are good study candidates as CKD biomarkers.

Regarding our results, although the levels of the five selected miRNAs are increased in the whole blood of mice fed adenine, only miR-542-3p, miR-748-3p, miR-361-3p, and miR-30c-2-3p correlate with parameters of renal function, particularly urea nitrogen plasma concentration. However, although this correlation was statistically significant, the best correlation coefficient obtained was 0.66. In summary, the analysis of the circulating levels of the miRNAs that modulate ILK expression does not add any value as a biomarker to the direct measurement of ILK in circulating mononuclear cells. Interestingly, as shown in Table 2 of the Results section, ILK deletion prevented the increased expression of miR-758-3p, miR-361-3p, and miR-30c-1-3p in the whole blood of adenine-fed mice, suggesting that ILK itself could regulate expressions of these miRNAs as an autoregulatory mechanism. Yuan D et al. described similar results in ovarian cancer by demonstrating that ILK silencing substantially increased the expression of four miRNAs (miR-15a-5p, miR-29c-3p, miR-30a-5p, and miR-200a-3p) [56].

## Conclusions

Altogether, our study demonstrates that ILK content in circulating mononuclear cells is indeed a rather good biomarker of renal and vascular tissue damage in an experimental model of CKD. To provide a clinical utility to this finding, additional approaches are needed to confirm it in humans and, especially, to assess the potential of ILK as a biomarker of CKD progression and/or CKD-associated vascular damage. Due to the numerous etiologies of CKD and the complex interactions of the multiple pathophysiological processes involved, a panel of biomarkers (and not just a single one) including ILK measurement could be necessary to improve this predictive ability.

## Data Availability

No datasets were generated or analysed during the current study.

## Abbreviations

CKD: Chronic kidney disease
cKD-ILK: ILK conditional-knockdown
CVD: Cardiovascular disease
ECM: Extracellular matrix
GFR: Glomerular filtration rate
ILK: Integrin-linked kinase
PBMCs: Peripheral blood mononuclear cells
RC: Renal cortex
RT-qPCR: Reverse transcription–quantitative polymerase chain reaction
WT: Wild-type
